# Aesthetic and Occlusal Rehabilitation Using a Telescopic Denture

**DOI:** 10.7759/cureus.7402

**Published:** 2020-03-25

**Authors:** Ang Yee, Goo Chui Ling

**Affiliations:** 1 Restorative Dentistry, The National University of Malaysia, Kuala Lumpur, MYS; 2 Restorative Dentistry / Prosthodontics, The National University of Malaysia, Kuala Lumpur, MYS

**Keywords:** prosthodontics, telescopic, partial denture, occlusion

## Abstract

Rehabilitating the occlusion of a patient with multiple missing posterior teeth may be challenging, especially when the remaining teeth are malaligned with loss of occlusal vertical dimension. A telescopic denture can be an excellent treatment alternative. In this case, the patient requested an aesthetic maxillary denture with no visible metal clasps when smiling. Hence, two telescopic crowns were placed on the anterior abutment teeth serving as the retentive components of the maxillary cobalt-chromium removable partial denture. Additional retention was obtained from the posterior abutment teeth. The patient was satisfied with the final restored occlusion and appearance.

## Introduction

There are numerous treatment options available for patients who require replacement of multiple missing teeth. In cases where only a few malpositioned teeth remain in the arch, removable partial dentures (RPD) or implant-supported prosthesis were usually the alternatives offered [1]. RPD is a cost-effective and acceptable treatment modality in replacing long edentulous spans. A telescopic denture uses the existing abutment teeth as retainers where these additional attachments serve to increase the retention and stability of the prosthesis [2]. A telescopic denture is defined as “an overdenture which is a dental prosthesis that covers and is partially supported by natural teeth, natural tooth roots, and/or dental implants” [3]. The term telescopic denture refers to the type of prosthesis that includes double crowns as retainers or attachments. These retainers consist of two crowns; primary or inner crown which is cemented to the abutment and secondary or outer crown which is attached to the denture. Many other names are used to describe similar types of prostheses such as a hybrid removable denture, an overlay prosthesis, a Marburg double crown system, etc. [4]. The purpose of this article is to present a clinical case in which the telescopic denture was fabricated on the maxillary arch to improve aesthetics and mastication. A short review of the laboratory aspects is discussed as well.

## Case presentation

**Figure 1:**
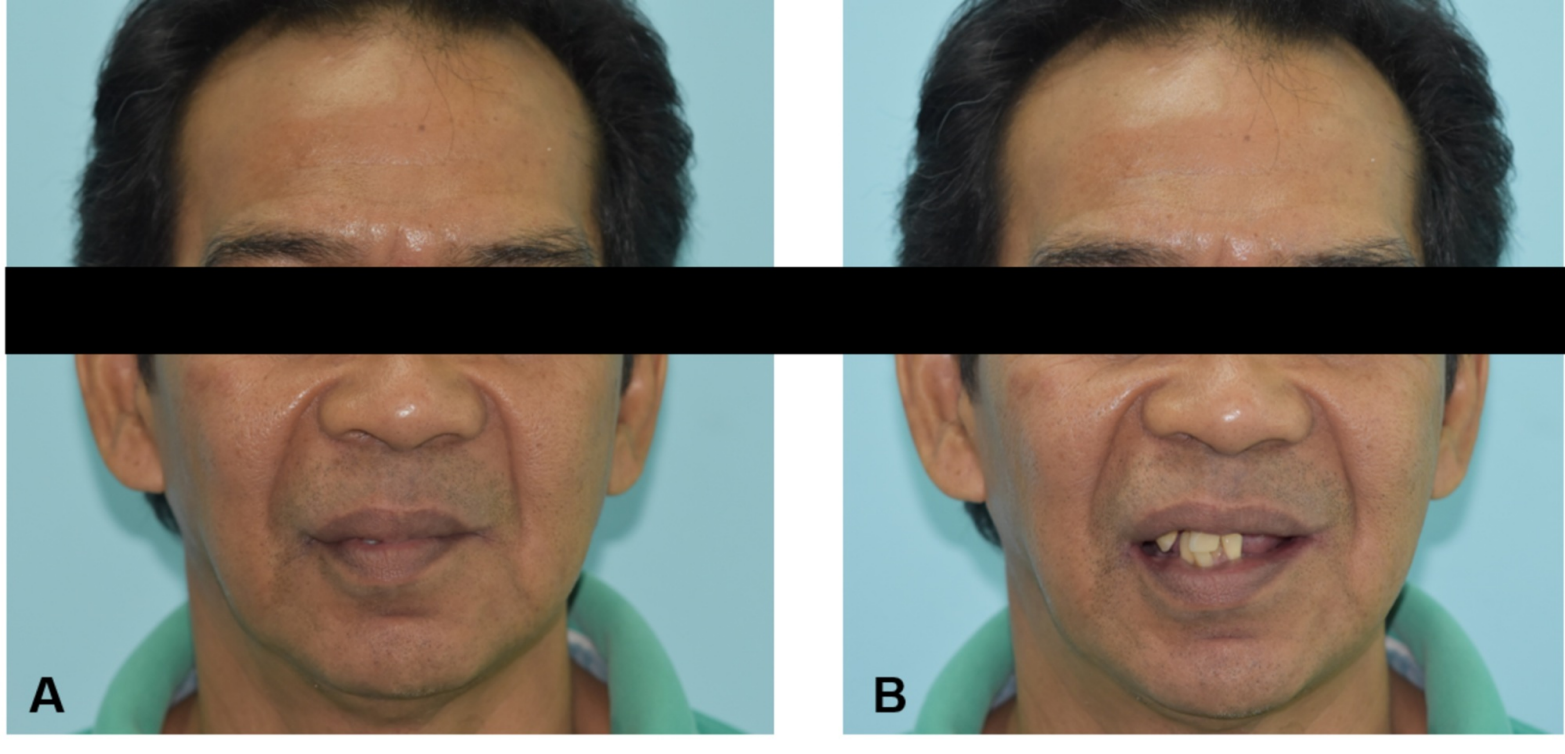
Extraoral view (A) Asymmetrical lips with lack of lip support, (B) Patient with average smile line

A systematically healthy, 51-year-old male requested for a set of dentures to replace his missing teeth. He had multiple teeth extracted over the past six years and claimed that they were non-restorable. He never had any form of replacement during his period of edentulism. He had difficulties in chewing, as only one upper tooth was in contact with the opposing teeth. He wished to have a set of dentures that can improve his chewing ability and provide satisfactory aesthetics without having any visible metal wires or clasps. Extraoral examination revealed asymmetrical lips with lack of lip support (Figure 1).

**Figure 2:**
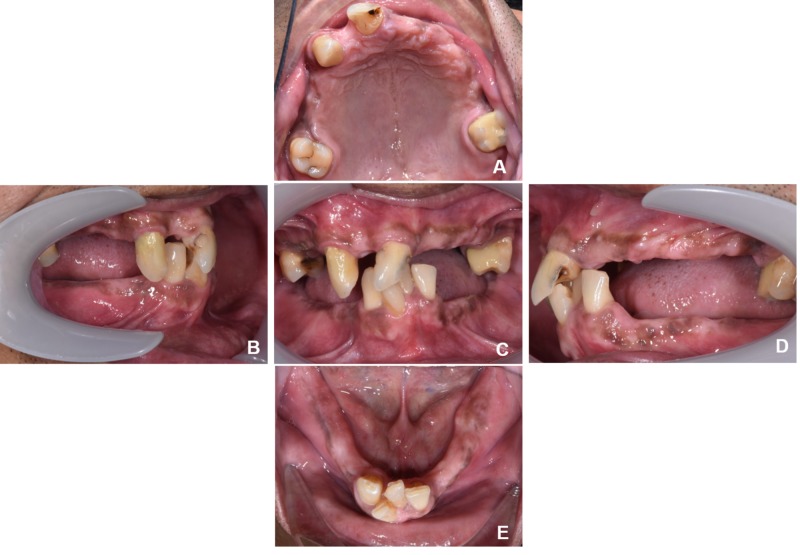
Initial intraoral photographs (A) Maxillary occlusal, (B) Right buccal, (C) Frontal, (D) Left buccal, (E) Mandibular occlusal

**Figure 3:**
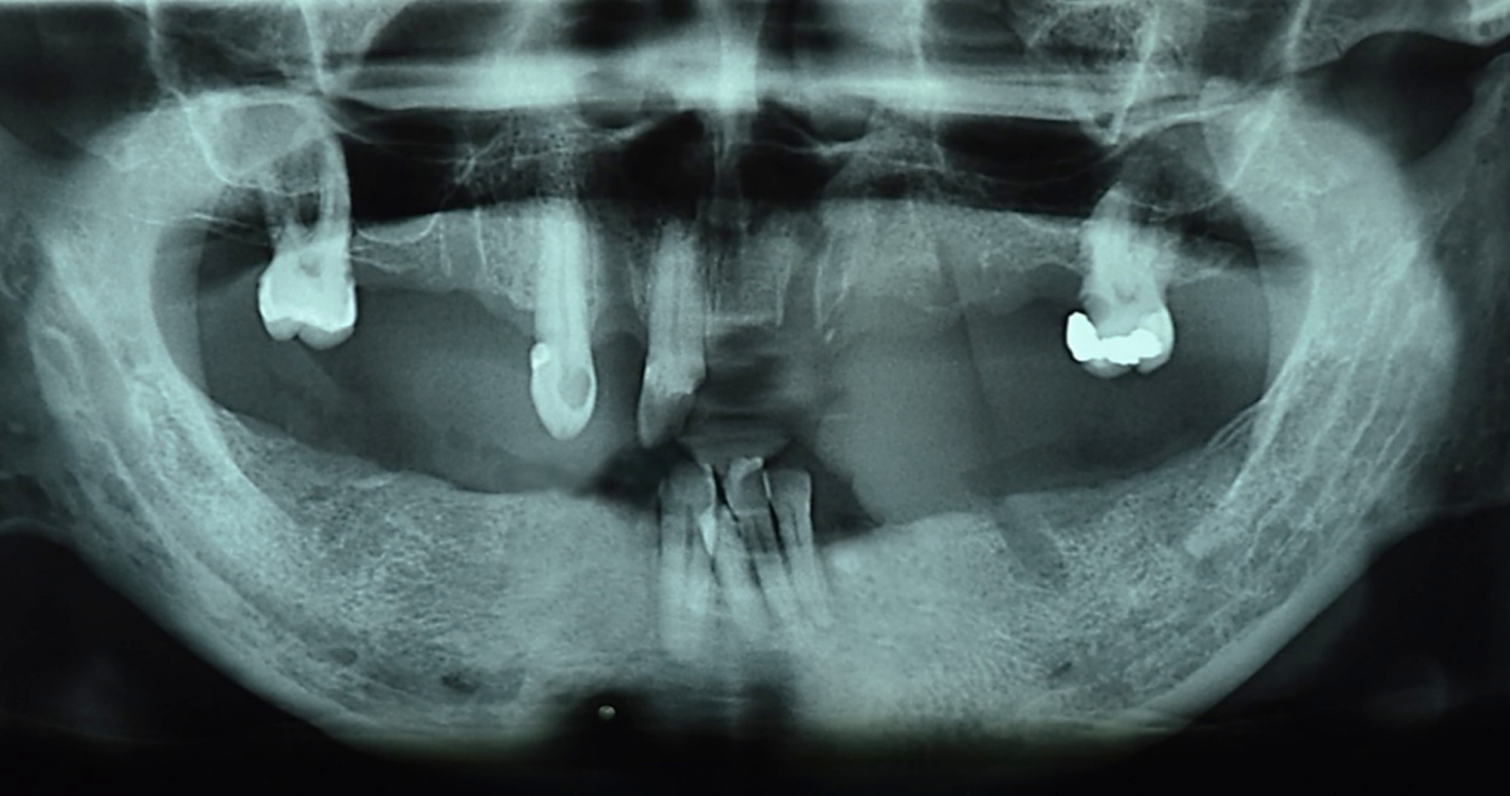
Dental panoramic radiograph

**Figure 4:**
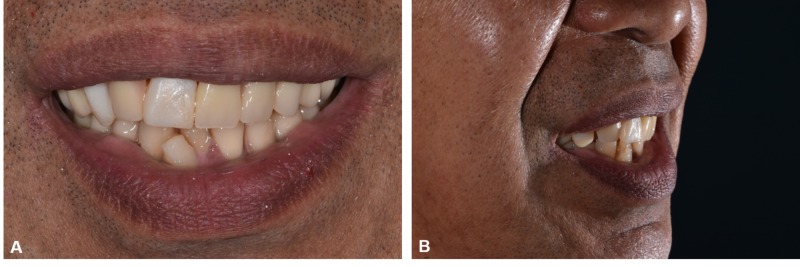
Stabilization phase (A) Interim dentures delivered but with canted midline, (B) Corrected vertical dimension of occlusion (VDO)

The existing maxillary teeth were teeth 17, 13, 11, and 26; the existing mandibular teeth were 31, 41, 42, and 43. Initial intraoral views and the dental panoramic radiograph were presented in Figure 2 and Figure 3, respectively. The vertical dimension of occlusion (VDO) was collapsed with a freeway space of 6 mm. The only occluding teeth were 11 with 41 and 42. Tooth 13 and 11 were diagnosed with asymptomatic irreversible pulpitis with asymptomatic apical periodontitis and were indicated for non-surgical endodontic therapy. Secondary caries without pulpal involvement was noted on tooth 26 and the tooth was eventually restored with a milled crown. During the provisionalization phase, an interim acrylic maxillary and mandibular dentures were issued to restore and test the increased vertical dimension (Figure 4).

**Figure 5:**
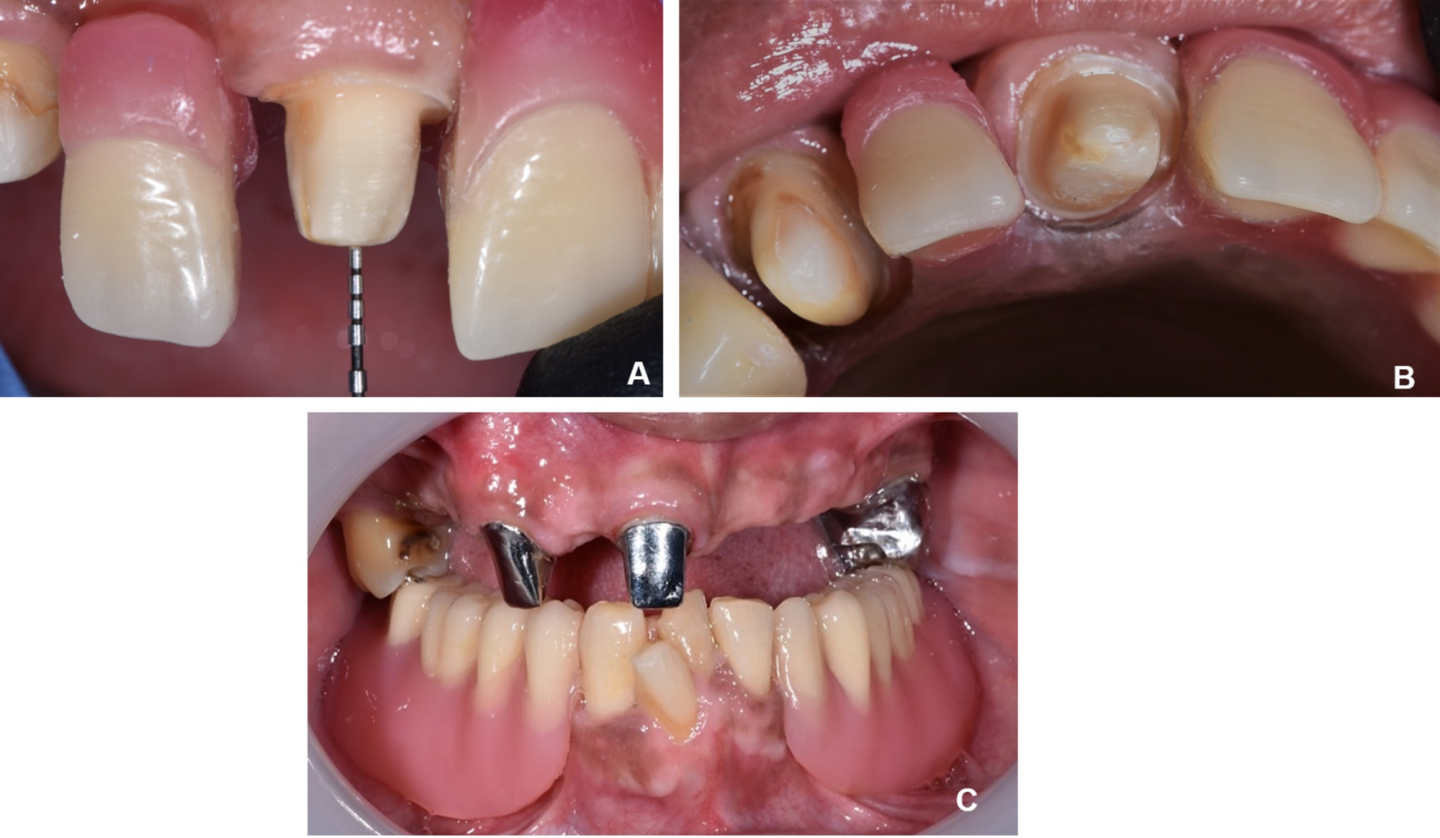
Telescopic crowns of 11 and 13 (A, B), Guided preparation (C) after cementation

**Figure 6:**
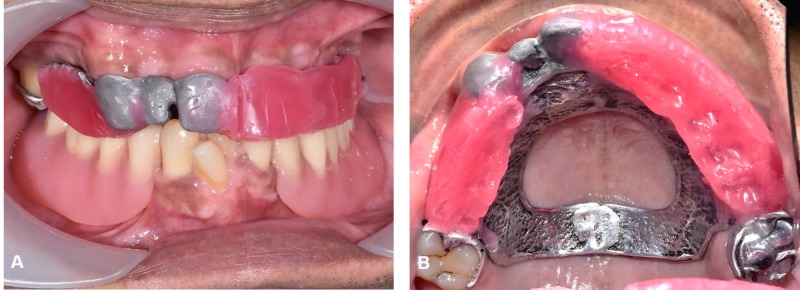
Framework try in with MMR recorded (A), Frontal view, (B) Occlusal view MMR: Maxillo-mandibular relationship

A diagnostic wax-up denture was used as a guide during the preparation so to achieve adequate tooth reduction (Figures 5a-5b). Later, the telescopic crowns with parallel mesial, distal, and labial surfaces were placed on teeth 13 and 11 (cobalt-chromium) (Figure 5c). The final impression for the telescopic denture was taken with a light body and regular body polyvinylsiloxane impression material. The telescopic cobalt-chromium framework tried in with satisfactory retention and stability. Maxillo-mandibular relationship (MMR) was recorded in centric relation (Figure 6).

**Figure 7:**
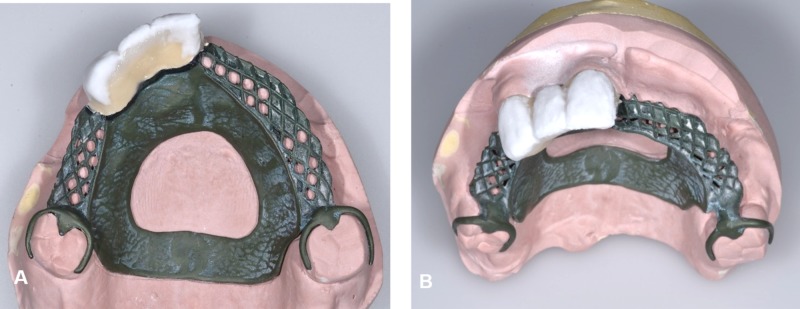
Porcelain layering at denture teeth 11, 12, and 13 prior to acrylic teeth arrangement and processing

**Figure 8:**
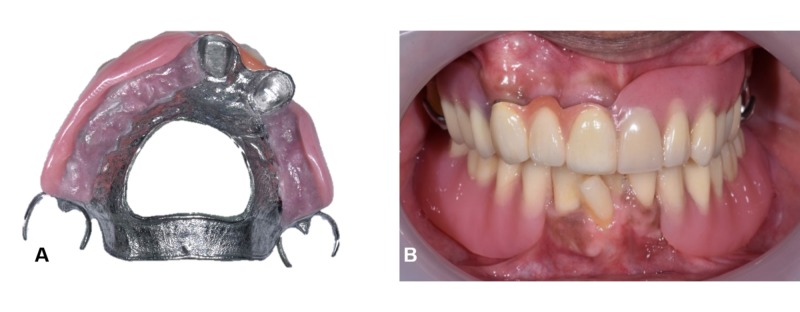
(A) Maxillary cobalt-chromium telescopic retained denture after processing (B) Denture insertion

**Figure 9:**
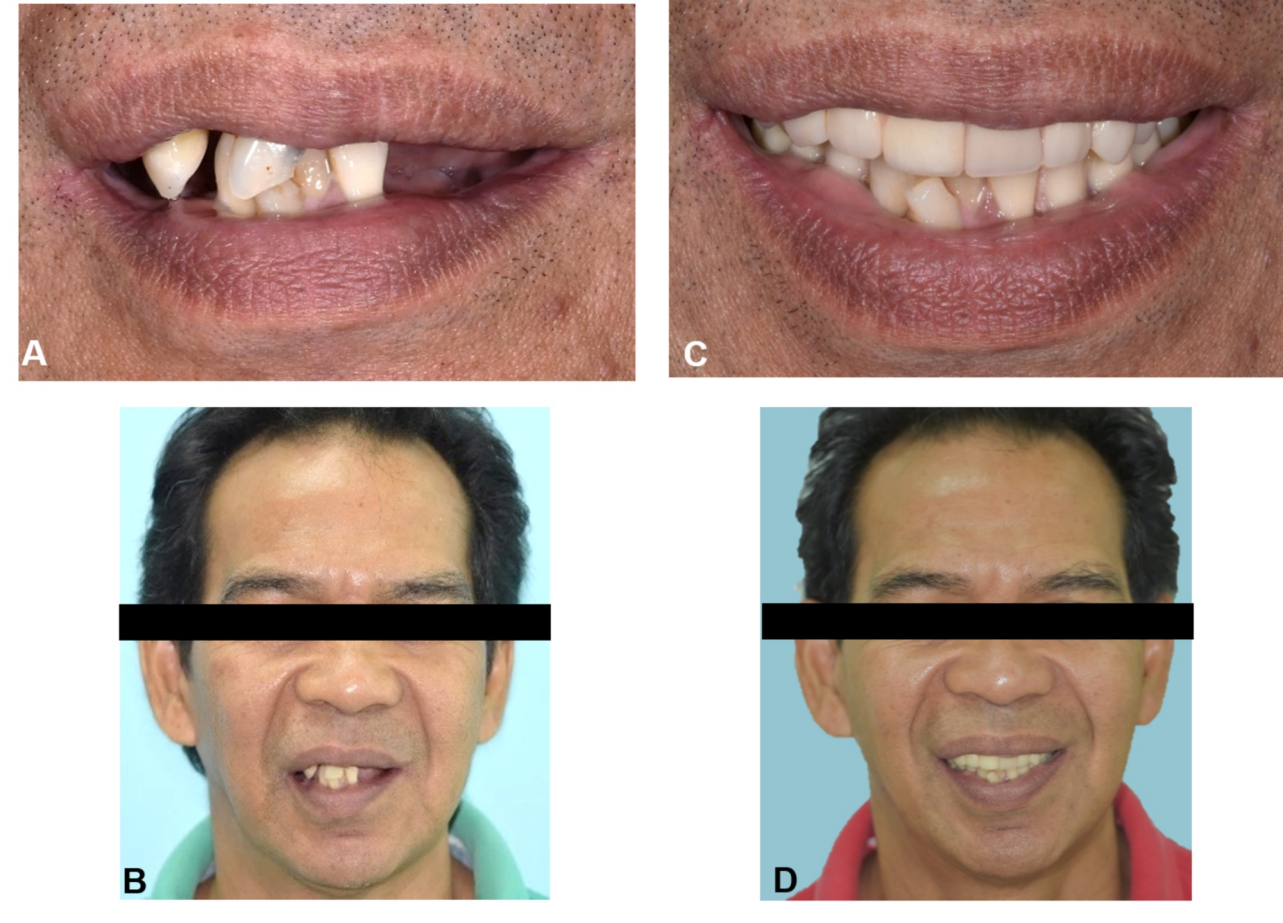
Pre- and postoperative photographs (A, B), Initial (C, D) Follow-up six months

Acrylic teeth were set up and tried in to assess the occlusion and aesthetics. Bilateral group function occlusion was achieved upon right and left excursion and even contacts on anterior prosthetic teeth during protrusive movements. A putty index was fabricated over the labial surface of the arranged acrylic teeth, acting as a template to ensure similar teeth arrangement after porcelain placement. The porcelain was layered over the area of teeth 11, 12, and 13 of the telescopic denture (Figure 7) using A3, A2, and transparent incisal feldspathic powder (IPS InLine ®, Ivoclar Vivadent, Schaan, Liechtenstein) and fired in the porcelain furnace (Programat P500, Ivoclar Vivadent). After final glazing, acrylic teeth were re-arranged following the putty index, and the denture was processed accordingly. The maxillary denture was delivered thereafter, and the patient was satisfied with both the aesthetic and functional outcome of the rehabilitation (Figure 8). A follow-up appointment revealed satisfactory oral hygiene and prosthesis maintenance. The mandibular denture was maintained in acrylic, as the patient was keen for implant placement in the near future when he had sufficient funds. Pre- and postoperative six months 'comparison is shown in Figure 9.

## Discussion

A telescopic denture is indicated when a few unfavorably distributed abutment teeth remained within the arch [5]. In this case, both the anterior abutment teeth 11 and 13 were labially tilted in a Class II relationship. Prescribing crowns may improve the angulation of the abutment teeth, but clasps placement was still mandatory on these abutment teeth to provide adequate retention and resistance of the RPD. In addition, the anterior abutment teeth were both extensively carious and required non-surgical root canal therapy. Hence, the abutment teeth could undergo more tooth reduction to cater to both the primary telescopic coping and secondary telescopic denture without risking the vitality of the abutment teeth [6]. With inner copings designed parallel to the proximal surface of the posterior teeth, a single path of insertion was achieved. Unlike extra-coronal precision attachments, these telescopic abutments were easily accessible, allowing effective home care and oral hygiene maintenance [7]. The position of the upper posterior abutments was on par with the design of the partial denture, therefore, it was not taken into account as telescopic abutments. In this case study, the occlusal scheme adopts bilateral group function occlusion so as to have even distribution upon left and right excursive movements. In addition, the uniform occlusal contacts play an important role, as it opposed the future implant-supported prosthesis.

Many double-crown systems have been reported in the literature. The first telescopic crown was patented by Dr. J. B. Beers in 1873 and later improvised by Langer (1980) who categorized them into three systems [8]. Cylindrical-shaped inner crowns provided remarkable retention and aesthetics in the marginal area. However, such crowns were difficult to fabricate, and the constant friction led to an increase in wear rate [9]. Conical shaped crowns with 6° tapering were widely used, as they were less harmful to the abutment teeth and supporting tissues. However, they were not as retentive as cylindrical crowns. Another telescopic coping described was resilient crowns where only the cervical half conformed to the cylindrical shape. The authors claimed that this design harmonized with the tissue elasticity, had better occlusal forces distribution, and, hence, increased the survival rates of the abutment teeth [10]. However, in a retrospective study, the survival of double-crown-retained RPD seemed to favor telescopic cylindrical design over conical and resilient crowns with a 90% success rate after seven years [11]. Only 78.5% of conical and resilient crowns survived. Henceforth, the majority of the surfaces of the inner copings, in this case, were made parallel with the determined path of insertion to provide the necessary retention.

Retention of the telescopic denture also relies heavily on the frictional surfaces. The components used for the inner crowns and secondary denture should have high shear strength and resistance to wear rates. An in-vitro study reported that telescopic crowns made of a non-precious metal offered better retention forces when compared with high noble metal or zirconia [12]. Both cobalt-chromium inner telescopic crowns and cobalt-chromium RPD provides retention forces as high as 12.5N as compared with gold inner crowns (7.4-9.6N). Another study comparing the telescopic denture to RPD retained via precision attachment and to another group with RPDs retained with conventional Aker’s clasps found that telescopic dentures had significantly higher homogenous occlusal force distributions among the abutments when compared to the other two groups. Hence, it was concluded that telescopic dentures provided the optimum support to the edentulous ridge and were able to prevent unwanted torque forces on the abutment teeth [13].

Porcelain layering over the cobalt-chromium framework demonstrated high fracture strength and excellent aesthetics but may be difficult to repair if a complication such as porcelain chipping occurred. Shade matching of the porcelain build-up with the adjacent acrylic teeth was also challenging in this case. An alternative suggested was the usage of composites as the veneering substrate over the framework but the fracture resistance and wear rates were questionable, as no long-term evidence is available for such a method [14]. 

Survival of telescopic retained RPD (T-RPD) was 100% after 5 years [15]. There were no statistical differences found between conventional RPD (94.5%) and T-RPD but complications raised from conventional RPD were more difficult to rectify with higher periodontitis and caries rate. Loss of cementation of the primary crowns was the commonest complication in T-RPD which could be easily handled clinically. In cases where abutment teeth served as telescopic retainers are lost or extracted, the denture could still function as usual without compromising the occlusion and aesthetics. The inner surface of the RPD replacing the abutments can simply be filled up with composite.

## Conclusions

A telescopic denture can be considered a viable treatment option for patients with unevenly distributed and/or malaligned abutment teeth within the arch. These RPD can easily rectify the aesthetics and possible retention problem that was commonly seen in conventional RPD. Besides, long-term maintenance of oral hygiene is relatively simple as compared to RPDs utilizing precision attachment systems.
